# Epileptic Electroencephalography Profile Associates with Attention Problems in Children with Fragile X Syndrome: Review and Case Series

**DOI:** 10.3389/fnhum.2016.00353

**Published:** 2016-07-12

**Authors:** Benjamin Cowley, Svetlana Kirjanen, Juhani Partanen, Maija L. Castrén

**Affiliations:** ^1^Brain Work Research Centre, Finnish Institute of Occupational HealthHelsinki, Finland; ^2^Cognitive Brain Research Unit, Cognitive Science, Institute of Behavioral Sciences, University of HelsinkiHelsinki, Finland; ^3^City of Helsinki Education DepartmentHelsinki, Finland; ^4^Department of Clinical Neurophysiology, University Hospital of HelsinkiHelsinki, Finland; ^5^Faculty of Medicine, Physiology, University of HelsinkiHelsinki, Finland; ^6^Autism Foundation in FinlandHelsinki, Finland

**Keywords:** fragile X syndrome, electroencephalography, clinical case series, endophenotype, attention deficit disorder, neurofeedback

## Abstract

Fragile X syndrome (FXS) is the most common cause of inherited intellectual disability and a variant of autism spectrum disorder (ASD). The FXS population is quite heterogeneous with respect to comorbidities, which implies the need for a personalized medicine approach, relying on biomarkers or endophenotypes to guide treatment. There is evidence that quantitative electroencephalography (EEG) endophenotype-guided treatments can support increased clinical benefit by considering the patient's neurophysiological profile. We describe a case series of 11 children diagnosed with FXS, aged one to 14 years, mean 4.6 years. Case data are based on longitudinal clinically-observed reports by attending physicians for comorbid symptoms including awake and asleep EEG profiles. We tabulate the comorbid EEG symptoms in this case series, and relate them to the literature on EEG endophenotypes and associated treatment options. The two most common endophenotypes in the data were diffuse slow oscillations and epileptiform EEG, which have been associated with attention and epilepsy respectively. This observation agrees with reported prevalence of comorbid behavioral symptoms for FXS. In this sample of FXS children, attention problems were found in 37% (4 of 11), and epileptic seizures in 45% (5 of 11). Attention problems were found to associate with the epilepsy endophenotype. From the synthesis of this case series and literature review, we argue that the evidence-based personalized treatment approach, exemplified by neurofeedback, could benefit FXS children by focusing on observable, specific characteristics of comorbid disease symptoms.

## Introduction

Fragile X syndrome (FXS) is the etiology of autism in 2–6% of all children diagnosed with autism spectrum disorder (ASD) (Dölen and Bear, 2009). FXS is also the most common inherited cause for intellectual disability ranging from mild to severe (Fisch et al., 2002). The phenotype of FXS includes distinct behavioral features that often disturb the normal daily life (Smith et al., 2012; Kidd et al., 2014). The most common behavioral problem in children with FXS is hyperactivity. Attention deficit hyperactivity disorder (ADHD) is diagnosed in approximately 70–80% of individuals with FXS (Baumgardner et al., 1995) whereas between 30 and 50% of individuals with ASD manifest ADHD particularly at pre-school age. FXS children who display hyperactivity tend to improve with age, and hyperactivity usually resolves by late adolescence, but attention problems often persist. A subpopulation around 30% of FXS individuals meet the criteria for ASD regarding communication difficulties, such as gaze avoidance, stereotypic and repetitive behavior, sensory abnormalities, and social anxiety (Hagerman et al., 2010). Aggressive behavior is more often problematic in adolescence and adulthood than in childhood and it can be difficult to handle. Obsessive-compulsive behavior is a common problem in FXS and it involves both children and adults. Epilepsy is found in 4–44% of FXS individuals (Kluger et al., 1996; Sabaratnam et al., 2001; Berry-Kravis, 2002; Louhivuori et al., 2009). Thus, symptomology arising from comorbidities may in fact be a considerable contributor to everyday distress for FXS individuals and difficulty for their caretakers.

Nowadays, no specific therapy is available for FXS. Nevertheless, successful treatment of comorbidities could alleviate symptoms non-specific to FXS, facilitating quality of life. There are various interventions that can be helpful and usually different interventions work synergistically (Hagerman et al., 2009). The responses to drug treatment vary and individual responses are sometimes unpredictable, particularly at young ages. Furthermore, effects of drug interventions in the immature brain are not well-understood, emphasizing the need for new effective treatment approaches without side-effects (Frye et al., 2013). The FXS population is quite heterogeneous with respect to comorbidities, which implies that: (a) the approach to each case should be personalized using validated methods; and (b) it may be practical to not (or not only) rely on standard treatments, but also to include non-drug treatments which can be tailored to each case. Personalized treatment emphasizes heterogeneity within a given disorder, relying on biomarkers or endophenotypes to guide different treatments, and can also help the subgroup of non-responders to traditional treatments. Johnstone et al. (2005) have argued in support of the personalized treatment approach for neuropsychiatric syndromes, suggesting “increased clinical benefit by considering the patient's neurophysiological profile.” They suggest that the application of quantitative electroencephalography (qEEG) analysis can help improve the objectivity of prescriptions for psychoactive medication, and the specificity of non-drug treatments such as neurofeedback (NFB). They suggest that treatments of many kinds can be guided by EEG profiles which are “manifestations seen between genome and behavior” that they term “intermediate” EEG endophenotypes. The approach of linking genes and behavior via EEG has since been pursued in diverse fields; as Porjesz and Rangaswamy state “brain oscillations provide a rich source of potentially useful endophenotypes for psychiatric genetics” (Porjesz and Rangaswamy, 2007).

Our motivating hypothesis is that: EEG endophenotypes as described in the literature are evident in FXS children and can therefore guide personalized treatment and improve disease understanding. We report a longitudinal case series of children diagnosed with FXS, including clinically-described EEG profiles. We collate the comorbid EEG symptoms in this case series, and relate them to the literature on EEG endophenotypes and associated NFB treatment options (Johnstone et al., 2005). Synthesizing the insights from the case series and literature review, we observe a relation between epileptiform-symptoms and EEG-indications of attention problems. Thus, the data suggest that the evidence-based personalized treatment approach, exemplified by NFB, could benefit FXS cases by focusing on observable, specific characteristics of comorbid disease symptoms.

## Materials and methods

Altogether 11 Finnish children (ten males and one female, identified herein by numbers 1–11: female case id = 9; male cases 1–8, 10–11) were included in the case series. Cases were selected from among a total of 23 children diagnosed with FXS at Kuopio University Hospital in a 10 years period. Selection criteria were: (i) the presence of EEG recordings, of which there were 12 cases; (ii) the presence of EEG abnormality, true for 92% (11 of 12). The children without any EEG, or without EEG abnormality, are not reported here.

All subjects participated voluntarily in the experiment, and a written informed consent was obtained from a parent of the child. The study was approved by the local Ethics Committee, and followed the guidelines of the Declaration of Helsinki. Since some children were measured at multiple times, we report the range of age at measurement, as 1–14 years, mean = 6 years. The longest gap between two consecutive measurements for one child was 7 years; the shortest was 4 months.

Case data reported here are based on the longitudinal clinically-observed reports of attending physicians, for comorbid symptoms including awake and asleep EEG profiles. Symptoms of comorbidities, including attention and motor problems, and epilepsy, were coded as binary variables to indicate presence or absence. See Table 1 for details.

**Table 1:** **Abridged findings from clinical observation notes for waking EEG and comorbidities**.

**id**	**#**	**Age**	**Abn**.	**EEG findings**	**AP**	**MP**	**Epi**.	**Drug**
1	1	41	2	Occipital: strong rhythmical alpha activity 6–8 Hz at 100 μV, decreases during eyes-open; Slow wave activity: increases twice, probably due to decrease in alertness; Paroxysmal transients: spike-slow wave components and sharp waves, spread frontal to convex, irritable cortex	0	0	0	N
	2	45	2	Occipital: strong rhythmical 7–8 Hz alpha-activity at 100 μV; Slow wave activity: increases with decreased alertness	1	0	0	N
2	1	24	1	Occipital: strong rhythmical activity 5.5–6 Hz at 140 μV, decreases when eyes are opened; Beta: some mild activity interfered with other activity	0	0	1	N
3	1	40	2	Occipital: strong symmetrical rhythmical activity 7–8 Hz at 70 μV; Paroxysmal transients: right (occasionally also left) centro-parietal spikes and spike-slow wave activity, reflected to dorsal temporal region, irritable cortex; Slow wave activity: increased in the same areas	1	0	0	N
	2	51	2	Occipital: strong rhythmical theta activity 6–7 Hz; Paroxysmal transients: right (occasionally also left) occipital spike-slow wave activity; Spike-slow wave components are seen to spread over convexity	1	0	0	N
	3	61	2	Occipital: strong rhythmical symmetrical activity 8–9 Hz at 70 μV; Paroxysmal transients: frontal and occipital spikes and spike-slow wave activity	1	0	1	V
	4	68	2	Occipital: 8 Hz activity; Paroxysmal transients: left occipital spikes and spike-slow wave activity; Right centro-parietal independent irritable activity	1	0	1	V
4	1	60	2	Occipital: rhythmical 7–8.5 Hz activity; Slow wave activity: normal diffuse; Paroxysmal transients: right dorsal temporo-parietal focal spikes, blinking increases intensity	1	1	0	N
	2	68	2	Occipital: rhythmical 6.5–8.5 Hz at 120 μV activity, slightly left-asymmetric; Slow wave activity: strong diffuse; Paroxysmal transients: spike-slow wave polymorphic activity in delta-range in right occipital areas, on the left theta	1	1	0	N
5	1	12	1	Occipital: strong rhythmical 4.5 Hz activity, delta asymmetry, higher amplitude on the right; Delta, theta: strong activity on the background; Beta: in the frontal region;	0	1	0	N
6	1	60	1	Slow wave activity: right fronto-temporal bilateral delta frequency discharges; Paroxysmal transients: left centro-temporal small sharp waves and spikes; no abnormalities	0	0	1	V
7	1	12	0	Occipital: rhythmical symmetrical 5 Hz activity; Beta: low in all channels; Slow wave activity: diffuse background activity, appropriate to the age	0	0	0	N
	2	21	0	Occipital: rhythmical 5–6 Hz at 140 μV activity; Slow wave activity: no change	0	0	0	N
	3	36	0	Occipital: rhythmical 5–6 Hz at 130 μV activity; Beta: normal in all channels; Slow wave activity: no change	0	0	0	A
	4	72	1	Occipital: rhythmical symmetrical slow activity 5.5–7 Hz at 150 μV; Slow wave activity: diffuse background activity increases	0	0	1	C
8	1	81	2	Occipital: labile broken rhythm 6.5–9 Hz; Theta: diffuse activity in excess; Beta: interfered with other activity; Paroxysmal transients: right temporo-parietal intermittent spikes; spikes and 2–3 Hz slow waves spread over convexity, irritable cortex	0	1	1	C
	2	132	2	Occipital: labile broken symmetrical rhythm 8–9 Hz at 60 μV; Slow wave activity: in excess; Paroxysmal transients: right frontal slow waves. Irritable cortex not observed	0	1	1	C
9	1	85	1	Occipital: rhythmical 8–10 Hz at 160 μV activity; Delta: dorsal region high-amplitude transients; Theta: diffuse, appropriate to the age Beta: especially in the frontal regions;	0	1	0	N
	2	168	0	Occipital: rhythmical 8–9 Hz at 90 μV activity; Beta: normal Paroxysmal transients: individual transients;	0	1	0	N
10	1	63	2	Occipital: rhythmical 7–8 Hz at 80 μV activity; Beta: low in all channels; Paroxysmal transients: left dorso-temporal spikes and spike-slow wave activity, reflected to the right and occasionally transient over convexity, irritable cortex	1	1	0	C
11	1	59	2	Occipital: rhythmical 6–8 Hz at 100 μV activity; Paroxysmal transients: right occipital delta transients; centro-temporal focal spikes	0	0	0	N

*Columns, from left to right, are: subject id; number of the measurement; age (in months at measurement); estimate of EEG abnormality (0 = normal, 1 = mild, 2 = abnormal); EEG findings; AP, attention problems (0 = not observed, 1 = present); MP, motor problems (0 = not observed, 1 = present); Epi., epilepsy symptoms (0 = no, 1 = yes); drug treatment (no long-term treatment = N, valproate = V, carbamazepine = C, antibiotics = A)*.

EEG recordings were performed as clinically required: for both waking and sleeping protocols, across one or more measurement points. Clinical EEG experts assessed the recordings for markers of abnormality and the recordings were coded at each observation point for the present analysis, 0 = normal, 1 = mildly abnormal, 2 = abnormal.

To estimate the prevalence in the sample of endophenotypic patterns (also coded as binary variables—see Table 1), the clinical record of the EEG findings were compared with the literature on EEG endophenotypes (Johnstone et al., 2005). The estimate was based on comparison of verbal descriptions (not the normative database procedure). To analyze patterns in the group data, we calculated Pearson product moment correlation coefficient, two-tailed.

## Results

### EEG recordings

Clinician classification of the 11 cases by level of abnormality is given as follows:

- One girl (case 9) transitioned from “mildly abnormal” to “normal EEG,” after maturation from 7 to 14 years old.
- One boy (case 7) transitioned from “normal EEG” in three measurements over 1, 2, and 3 years old, to “mildly abnormal” after measurement at 6 years.
- One boy (case 8) transitioned from “abnormal EEG” at age seven to “mildly abnormal” age 11.
- Three boys (cases 2, 5, 6) were classified as “mildly abnormal.”
- Five boys (cases 1, 3, 4, 10, 11) were classified as “abnormal EEG.”

Waking EEG findings are described in abridged form in Table 1. There were two main patterns (endophenotypes) in waking EEG recordings, each with *N* = 7 (see Table 1). These include slowing of background rhythm (“diffuse slow” endophenotype) and epileptiform discharges (“epileptiform” endophenotype). Other less frequent findings include focal abnormalities, mixed fast and slow rhythms, excess beta, and generally low magnitudes.

Most of the correlations between EEG features and symptoms of comorbidity were only moderate and non-significant. The highest correlations were between attention problems and EEG abnormality (Pearson's *r* = 0.4, *ns*); and between attention problems and the “epileptiform” endophenotype (Pearson's *r* = 0.3, *ns*). The “diffuse slow” type did not correlate strongly with any behavioral comorbidities. Assessing the correlations *within* the set of EEG features, the “epileptiform” endophenotype is the clear main influence on EEG abnormality, Pearson's *r* = 0.9, significant at *p* < 0.00001, *df* = 19, *t* = 10.

Main findings from sleep EEG included that for two cases (Kluger et al., 1996; Dölen and Bear, 2009) the waking EEG abnormalities were no longer present; they were present in cases 2, 3, 5, 6, 8, 9, 10, 11 (case 4 not recorded in sleep) – see Table 2. For these cases, two main findings in sleep are that irritable cortex was observed to increase (case 3, 8), and spike or slow-wave discharges showed asymmetrically (case 2, 6, 6, 10).

**Table 2:** **Abridged clinical observations of sleep EEG (where performed, e.g., case id = 4 was not recorded at all, cases 1, 9 only at second measurement)**.

**id**	**#**	**age**	**Sleep EEG**
1	2	45	Normal sleep-EEG; When alertness is decreased the rhythmical activity of the back areas is desynchronized and slow wave activity is increased; During sleep sharp K-complexes, vertex-waves and sleep-spindles; the findings made in wake-EEG are not seen here
2	1	24	When the alertness is decreased the rhythmical activity of the back areas is desynchronized and diffuse theta and delta-activity can be seen; During sleep K-complexes, vertex-waves and sleep-spindles; Also left frontal (as compared to right) slow wave discharges (theta-delta, high in amplitude) are potentiated in sleep
3	1	40	Central vertex-potentials and sleep-spindles
	2	51	Abnormal sleep-EEG; Irritable findings are present and even increased during sleep and especially spike-slow wave components are seen spread over convexity
	3	61	Abnormal sleep-EEG; K-complexes; Irritable findings are increased during sleep
5	1	12	K-complexes and sleep-spindles; Occipital delta asymmetry, higher amplitude on the right
6	1	60	Especially during sleep left middle temporal small sharp waves and spikes
7	1	12	Slow-wave activity increase in sleep; During sleep K-complexes, vertex-waves and sleep-spindles
	3	36	During sleep vertex-waves
	4	72	Slow-wave activity increases in sleep; During sleep K-complexes, vertex-waves and sleep-spindles
8	1	81	Abnormal sleep-EEG; Irritable findings are increased in sleep (10 s each, with 10–20 s intervals); A lot of rhythmical delta activity (2.5 Hz) in the right hemisphere
9	2	168	Theta paroxysms and increase in diffuse theta during the decrease in alertness
10	1	63	During sleep left dorso-temporal spikes, vertex-waves and sleep-spindles
11	1	59	During sleep K-complexes, vertex-waves and sleep-spindles; Centro-temporal focal spikes

### Clinical observations

Clinical observations are described in abridged form in Table 1. All children with FXS displayed mild to moderate developmental delay that associated with difficulties in language development. Attention problems were observed in four FXS children (cases 1, 3, 4, 10). Symptoms of epilepsy including clinical seizures combined with EEG abnormalities were observed in five children (cases 2, 3, 6, 7, 8). Seizures that associated with fever and infections appeared at younger ages (1.5 and 2 years old in two FXS boys) than non-febrile seizures which were seen at the age of 4–5.6 years. In agreement with previous reports, epileptic seizures were complex partial or secondarily generalized. Furthermore, all five FXS males with epilepsy responded well to treatment with carbamazepine or valproate, which reduced EEG findings for three, and normalized the EEG for one boy. One FXS boy was treated with carbamazepine at age 5 years because of electrographic abnormalities that associated with restlessness and anxiety. The treatment alleviated his symptoms.

Based on the clinical reports of EEG, we estimated the prevalence of the EEG endophenotypes (Johnstone et al., 2005) for the 11 cases, see Table 3. The EEG observations of these 11 cases follow an expected pattern for the FXS, with a high prevalence of the “1.diffuse slow” and “7.epileptiform” endophenotypes. In the “diffuse slow” type (Johnstone et al., 2005), an increase in slow activity and a decrease in mean frequency of alpha indicate decreased activation level in brain which is often seen in pervasive developmental disorders, dementing illness, and other disorders of consciousness. Notably, the four FXS children who were noted to have problems with attention were classified to “epileptiform” type.

**Table 3:** **EEG endophenotypes (Johnstone et al., 2005), and prevalence in the sample population as estimated from clinical observations**.

**#**	**EEG biomarker**	**Case matches**
1	Diffuse slow activity, with or without low frequency alpha	1, 2, 3, 4, 5, 7, 11
2	Focal abnormalities, not epileptiform	6
3	Mixed fast and slow	5, 8
4	Frontal lobe disturbances	
5	Frontal Asymmetries	
6	Excess temporal lobe alpha	
7	Epileptiform	1, 3, 4, 6, 8, 10, 11
8	Faster alpha variants, not low voltage	
9	Spindling excessive beta	9
10	Generally low magnitudes (fast or slow)	10
11	Persistent alpha with eyes open	

## Discussion

The present results highlight the heterogeneity of the clinical phenotype as well as the EEG profile in FXS. Table 3 illustrates a mainly bimodal distribution of EEG endophenotypes across the cases; the primary modes are “diffuse slow” and “epileptiform.” Four cases shared these two endophenotypes. The correlations between abnormal EEG, “epileptiform” endophenotype and attention problems were marginally significant before multiple comparison correction^1^, indicating a trend that incidence and degree of epileptic EEG relates to attention problems in FXS. The phenomenon may also be related to the observation of abnormal attention-cued ERPs, specifically increased N1 amplitude and power to auditory standards compared with healthy controls (Castrén et al., 2003).

FXS is a monogenic syndrome that is caused by a mutation in the *FMR1* gene leading to the absence of the FMR1 protein. FMR1 protein is needed for normal maturation of synapses and neuronal circuit formation. Its absence results in hyper-excitability of neocortical circuits and alterations of network synchrony in brain of FXS mouse model (Gibson et al., 2008; Gonçalves et al., 2013). Similarly, defects of neuronal connections and disturbed inhibitory and excitatory balance have been implicated in infantile autism.

Genetic heterogeneity contributes to the FXS phenotype and incidence of co-morbidities, including ADHD which has a heterogeneous background. There is evidence that the serotonin transporter (5-HTTLPR) genotype modulates the aggressive, destructive, and stereotypic behavior of FXS individuals whereas monoamine oxidase A (MAOA-VNTR) polymorphisms correlate with the consumption of drugs that regulate serotonin reuptake (Hessl et al., 2008). In Finland, where fragile X chromosomes show a major haplotype, epilepsy was found to associate with polymorphisms in the gene for brain-derived neurotrophic factor in a subpopulation of FXS men (Louhivuori et al., 2009). Epileptic seizures in FXS show an age-related appearance in the childhood or young adulthood and are less common in adults. The developmental nature of epilepsy could reflect maturation deficits of the brain and be related to increased heterogeneity of the EEG profiles in childhood.

Epileptic seizures in FXS are usually easily treatable with traditional anti-epileptic drugs (AEDs) and the treatment is recommended based on several retrospective studies (Frye et al., 2013). However, the effects of AEDs on behavioral and cognitive symptoms in FXS and ASD have not been systematically studied. Our present finding, that the attention problems were associated with the epileptic EEG endophenotype, suggests that treatment helpful for seizures could be beneficial for the attention problems in FXS. There is evidence that certain AEDs could be appropriate to treat seizures of certain individuals with ASD (Frye et al., 2013), but there are no well-controlled clinical trials to support the effectiveness and efficacy of AEDs in ASD. Also, Frye et al. (2013) illustrate that all AEDs indicated for seizures in ASD have a considerable number of potential side-effects. For example, of the treatments for cases described here, valproate associates with hepatotoxicity, thrombocytopenia, hyperammonemia, and alopecia whereas carbamazepine associates with dizziness, ataxia, nausea, and hyponatremia. For NFB there are no side effects reported (Frye et al., 2013).

Ultimately the problem with AEDs is lack of specificity. Even in this small cohort sampled from a relatively homogenous population, heterogeneity of symptoms is high. No EEG endophenotype is dominant; rather the distribution is quite bimodal. The variation in disabilities is high. We propose that evidence from EEG data of FXS cases firstly indicates NFB training for specific relief of comorbid symptoms, and secondly provides a strong foundation for further studies to more fully characterize the neural profiles of the disorder at the whole brain level, by probing the relationship between brain and behavior using NFB. We elaborate these two arguments in the next two sections.

### EEG phenotypes and neurofeedback in FXS

NFB, also called EEG biofeedback, is operant conditioning of specific temporal, spatial and frequency features extracted from scalp-recorded electrical potentials (Lubar and Shouse, 1976). Literature supports the efficacy of NFB for children with ADHD (Arns et al., 2009, 2014; Micoulaud-Franchi et al., 2014), and with epilepsy (Sterman, 2000). The effect of NFB for ASD has not been so well-studied. Multiple individual case studies or case series have been reported, and a small number of group studies (Frye et al., 2013; Pineda et al., 2014). Coben et al. (2010) concluded a recommendation of “possibly efficacious,” though based on only two group studies. A more recent and systematic review by Frye et al. (2013) on treatments for seizures in ASD, found that NFB had a grade of recommendation of B for “seizures,” B for “behavioral and cognitive ASD symptoms,” and C for “ASD with seizures,” with no adverse effects reported^2^. They characterize NFB as “a safe treatment that uses operant conditioning to increase coherence between seizure prone and non-seizure prone brain regions.” An earlier review by Holtmann et al. (2011) suggested that, while “existing evidence does not support the use of [NFB] in the treatment of ASD,” it is possible that studies might be showing an improvement in comorbid ADHD symptoms, rather than core ASD symptoms. This latter point supports our thesis that NFB for FXS comorbidities is indicated.

Part of its value is that NFB can be personalized to suit the specific clinical presentation, provided that there is requisite theoretical and observational data to guide the personalization. Although earlier work (Lubar and Shouse, 1976; Monastra et al., 2005), including meta-analysis by Snyder and Hall (Snyder and Hall, 2006), has shown support for a single-trait model of ADHD (an elevated theta-beta ratio), others have argued that research results and clinical application should be interpreted in a more specific way (Arns et al., 2008). Hammond (2010) goes into this issue in detail, illustrating the heterogeneity in qEEG patterns associated with symptoms and discussing the requirements and need for qEEG analysis guided by normative databases. Johnstone et al. (2005) provide a review of such databases, along with a review of qEEG endophenotypes, which we have associated with FXS cases above.

EEG endophenotypes have been shown to have predictive value for disease assessment. For example, Fonseca et al. (Fonseca, 2008) found that ADHD children (*N* = 30) had significantly greater diffuse slow-frequency power than age- and sex-matched controls. Chabot et al. (1996) found that a qEEG-based discriminant function approach to identify ADHD from normal and learning difficulty children, had specificity and sensitivity around 90%. EEG endophenotypes are also predictive for treatment outcome, as shown by Arns et al. (2008) in a study of ADHD children (*N* = 49) on stimulants.

We observed two particular EEG endophenotypes in this case series. A strong relationship between the “epileptoform” endophenotype and the occurrence of attention problems suggest that the treatment of this endophenotype (described by e.g., Johnstone et al., 2005) may be indicated in FXS. In the suggested treatment approach (Johnstone et al., 2005), NFB training is targeted to reduce overall slow wave activity and increase sensory motor rhythm in the same site as the location of the seizure onset. This protocol aims to lower cortical hyper-excitability by reducing a key trigger for sharp waves.

### Endophenotype exploration in FXS

Our suggestion is to more fully characterize the neural profiles of FXS at the whole brain level, by probing the relationship between brain and behavior during NFB. The basic concept is to use NFB for neurophenomenology, as suggested by Bagdasaryan and Van Quyen (2013), and link neural activity to attention in a causally closed loop.

The motivation stems from the fact that FXS is *genetically* well-characterized, whereas e.g., ADHD is not. As Gevensleben et al. (2014) argue, efficacy of NFB is hard to assess without a proper model of specific effects of treatment. Further, Zuberer et al. (2015) argue that such a model should be tested by examination of the neurological data corresponding to operant conditioning progress, i.e., learning curves. However, in the neuropsychiatric disorders commonly treated by NFB, the etiology of the disorder is often not known, or unclear. This makes it hard to interpret the data.

The approach may be better supported by the well-characterized monogenic disease model such as FXS, which also appears to have clearly expressed EEG endophenotypes within the group. With a well-defined disease model, a clear endophenotype, and sufficiently well-prepared neurological and behavioral data on learning and expectations, there is an opportunity to accurately relate the treatment to the observed effects. The opportunity also meets a clinical need based on the estimation that in spite of the good supporting evidence for treating seizures and ASD, prevalence of use of NFB is less than 1% (Frye et al., 2013).

Gevensleben and colleagues describe two polar models for the application of NFB, the “conditioning and repairing” model and the “skill acquisition” model. Conditioning and repairing refers to normalization of a well-defined “specific neurophysiological deficit” by implicit learning, while skill acquisition refers to explicit learning of self-regulation, potentially by anyone regardless of neurological health. While FXS individuals certainly possess the specific neurophysiological deficit, the FXS condition itself is not a viable target for “normalization,” and the target of treatment is thus the complex range of comorbidities. As Gevensleben et al. points out “Complex attentional and social behaviors (encompassing different top-down and bottom-up mechanisms) rely to a larger extent on self-regulation skills and will not change to a clinically significant level due to distinct neurophysiological changes alone but have to be addressed on different levels. Neurophysiological changes must spread out beyond NFB-trained neuronal circuits and be accompanied by changes in cognitive-behavioral patterns to achieve enhanced self-regulation in complex environments.”

The research strategy we propose is to separate the treatment by contiguous conditions in a way that “normalization” training takes place before “self-regulation” training. A similar approach has been recently piloted (Cowley et al., 2016). The effects due to each condition can be observed in isolation and in the context of the well-defined theoretical model of NFB and a well-characterized disease model.

## Conclusion

In this paper we have described the clinical and EEG symptoms of 11 diagnosed FXS cases. We assessed the EEG symptoms in terms of the literature on EEG endophenotypes, and found that “epileptiform”-like symptoms are associated with attention problems in these cases. We then developed the argument that specific treatment for EEG symptoms is indicated.

The brain has an adaptive nature and ability to self-regulate when receiving feedback (Johnstone et al., 2005). This implies that the function of NFB to promote self-regulation (Mayer et al., 2013), (a non-specific effect of the operant conditioning paradigm), may further benefit treated FXS children's capacity to (co)operate in social and educational contexts. NFB is also marked by a relative ease and flexibility compared to medications, since the neurophysiological status is under constant assessment during treatment, and the protocols can be changed at any time to respond to the clinical development. Thus, we conclude that the need exists for a novel line of study, based on clinical trials of NFB for FXS comorbidities, with the opportunity not only to test the treatment efficacy but also to establish a more complete characterization of the behavioral and systems neuroscience of the disorder and related symptoms.

## Author contributions

BC conducted the analyses and co-wrote the draft; SK provided translations and contributed to the draft; JP analyzed the EEG data and reviewed the draft; MC co-wrote the draft.

## Funding

The work was supported by grants from Arvo and Lea Ylppö Foundation, Foundation for Pediatric Research, and Finnish Brain Research Foundation.

### Conflict of interest statement

The authors declare that the research was conducted in the absence of any commercial or financial relationships that could be construed as a potential conflict of interest.

## Footnotes

^1^Naturally at this sample size, *p*-value statistical testing is somewhat underpowered.

^2^The classification levels B, C are defined as follows (for full details of the scheme see Frye et al.): B - At least one study of type: “1b Prospective high-quality randomized controlled trial (RCT),” “2a systematic review (SR) of cohort (prospective, non-randomized) studies with homogeneity,” or “3a SR of case-control (retrospective) studies with homogeneity”; or two studies of type: “2b Individual cohort (prospective, non-randomized) study or low-quality RCT” or “3b Individual case-control (retrospective) study.” C - At least one study of type: “2b” or “3b”; or two studies of type: “case series or reports.”
